# Shigellosis Caused by CTX-M Type ESBL Producing* Shigella flexneri* in Two Siblings of Rural Nepal: First Case Report from the Country

**DOI:** 10.1155/2017/1862320

**Published:** 2017-02-21

**Authors:** Narayan Prasad Parajuli, Govardhan Joshi, Bashu Dev Pardhe, Jyotsna Shakya, Anjeela Bhetwal, Shreena Shakya, Roshan Pandit, Sumesh Shreekhanda Shrestha, Puspa Raj Khanal

**Affiliations:** ^1^Department of Clinical Laboratory Services, Manmohan Memorial Medical College and Teaching Hospital, Kathmandu, Nepal; ^2^Department of Laboratory Medicine, Manmohan Memorial Institute of Health Sciences, Kathmandu, Nepal; ^3^Kathmandu Center for Genomics and Research Laboratory (KCGRL), Kathmandu, Nepal

## Abstract

Shigellosis is an acute infectious disease characterized as severe bloody diarrhea (dysentery) and is accountable for a significant burden of morbidity and mortality especially in children under the age of 5 years. Antimicrobial therapy is required in the cases of severe dysentery associated with* Shigella*. However, emergence of multidrug resistant (MDR) strains of* Shigella* spp. over the last two decades has restricted the use of common therapeutic antimicrobials. In MDR strains, the third-generation cephalosporins have been used for the treatment, but, unfortunately, emerging reports of enzyme mediated *β*-lactam resistance among* Shigella* isolates from various parts of the world have greatly compromised the therapy of pediatric dysentery. In Nepal, drug resistant strains of* Shigella* spp. have been reported, but MDR and extended spectrum *β*-lactamase (ESBL) producing strains were previously unknown. Here, we report two* Shigella flexneri* isolates harboring ESBL genotype-CTX-M associated with acute dysentery in two siblings which were presented and treated in a tertiary care teaching hospital of Kathmandu, Nepal.

## 1. General Background

Shigellosis, caused by members of the bacterial genus* Shigella* [1], is an acute diarrheal disease primarily affecting poor, crowded communities that do not have adequate sanitation or clean water [2]. Globally,* Shigella* spp. are the most common cause of acute bloody diarrhea (dysentery) and are accountable for a significant burden of morbidity and mortality associated with diarrheal disease especially in children under the age of 5 years [3]. Clinically, shigellosis may range from mild self-limiting diarrhea to severe dysentery with frequent passage of blood and mucus, high fever, cramps, tenesmus, and dehydration [4]. Every year, about 125 million new cases of shigellosis occur in Asia alone, of which around 14,000 are lethal representing the burden of the disease in this region [5]. Among various serogroups,* Shigella flexneri*,* Shigella sonnei*, and* Shigella boydii* are predominant in developing countries, while* S. sonnei* is frequently reported from industrialized countries [6]. Antimicrobial therapy is required in the cases of severe dysentery associated with* Shigella* to reduce the duration of clinical illness, minimizing the complications, as well as prevent the dissemination of infectious cases [7, 8]. However, emergence of multidrug resistant (MDR) strains of* Shigella* spp. (i.e., resistance to more than two first-line oral drugs, such as ampicillin, cotrimoxazole, and ciprofloxacin) over the last two decades restricted the use of common therapeutic antimicrobials [9]. In MDR strains, the third-generation cephalosporins have been used for the treatment, but, unfortunately, emerging reports of enzyme mediated *β*-lactam resistance among* Shigella *isolates from various parts of the world have further compromised the pediatric therapy [7, 10]. In Nepal, drug resistant strains of* Shigella* spp. have been reported [11], but MDR and extended spectrum *β*-lactamase producing strains were previously unknown. Here, we report two* Shigella flexneri* isolates harboring ESBL genotype-CTX-M associated with acute dysentery in two siblings which were presented and treated in a tertiary care teaching hospital of Kathmandu, Nepal.

## 2. Description of the Cases

A six-month-old infant female was taken to the emergency department of Manmohan Memorial Medical College and Teaching Hospital, Kathmandu, Nepal, at 4:30 pm on 9 July 2016 with complaints of abdominal pain, frequent passage of loose, bloody, and frothy stool for the last 6 days. She did not have any history of fever, nausea, and vomiting. She belonged to the lower socioeconomic family and living in the earthquake affected remote area of Dhading district. They were using drinking water from nearby stream but have no history of eating anything unusual or unhygienic. On examination, she was conscious but lethargic and appeared pale. Her abdomen was soft with increased bowel sound but apparent hepatosplenomegaly was not noted. Chest was clinically clear. Her body temperature was 98°F, pulse rate was 115/minute, and respiratory rate was 30/minute. The patient was provisionally diagnosed as a case of acute diarrheal illness with suspicion of dysentery and immediate management was started. After necessary physical examination, blood and stool samples were collected aseptically for laboratory investigations, namely, complete blood count, blood chemistry tests, stool culture, and microscopy. The patient was then admitted to the pediatric unit for further treatment. Antimicrobial regimen of 1 g/24 hours of ceftriaxone and 500 mg/24 hours of metronidazole was initiated as empiric therapy for acute diarrheal illness along with zinc tablets (10 mg/24 hour), probiotic-bifilac (10 ml/24 hours), and oral rehydration solution (100 ml per stool episode).

## 3. Laboratory Findings

There was reduction in the hemoglobin concentration (Hb: 9.5%) but a normal (unremarkable) level of total leukocyte count with adequate cellular distribution (52% granulocytes and 48% lymphocytes). Other blood cell parameters and indices were found in the acceptable range. The clinical parameters of blood urea, electrolytes, and common liver enzymes were found normal. C-reactive protein by latex agglutination was also negative. Urine microscopy and chemical findings were normal. There were no remarkable changes observed in abdominal ultrasonography.

On stool microscopy, numerous leucocytes and erythrocytes along with cysts and trophozoites of amoeba* (Entamoeba *species) were observed. Further, the stool specimen was plated onto the Blood and MacConkey agar plates (Hi-Media Laboratories, Mumbai, India) and incubated aerobically at 37°C for 24 hours. After incubation, numerous nonlactose fermenting colonies were observed on MacConkey agar while pale colonies with no hemolysis were seen on Blood agar. These nonlactose fermenting colonies were picked and processed for biochemical characterization and antimicrobial susceptibility testing by Kirby Bauer disk diffusion method and result of susceptibility test was interpreted according to the CLSI guidelines for Enterobacteriaceae [12].

Biochemical results suggested that the isolate belongs to the genus* Shigella *and further serotyping was carried out using specific antisera (Denka-Seiken, Japan) and it was confirmed as* Shigella flexneri. *On the susceptibility testing, the isolate was found resistant to penicillins, macrolides, cephalosporins, fluoroquinolones, and trimethoprim-sulphamethoxazole group of antibiotics (Table 1). Therefore, we suspect the isolate to be presumptive ESBL producer and further characterization of ESBL was done by combination disk test. In this test, Ceftazidime (30 *μ*g) disks alone and in combination with clavulanic acid (Ceftazidime + clavulanic acid, 30/10 *μ*g) disks were applied onto a plate of Mueller Hinton Agar (MHA) which was inoculated with the strain and then incubated in ambient air for 16–18 hours at 35 ± 2°C. The isolate showed the increase of ≥5 mm zone diameter on combination disk compared to that of the Ceftazidime disk alone and was considered an ESBL producer [12] which was later tested for genotypes.

Despite continuous fluid replacement and intravenous therapy of cephalosporin (ceftriaxone) and metronidazole, apparent prognosis in the disease was not observed. On day four, the bacteriological report stating the presence of ESBL producing* Shigella flexneri* was received and the antimicrobial regimen was changed to Piperacillin tazobactam (2 gm/24 hour) and Metronidazole (450 mg/24 hour). Fortunately, after instituting the durataz (Piperacillin + tazobactam), the frequency of loose motion was dropped down gradually and clinical signs of abdominal discomfort were resolved. This therapy was continued for the next 5 days and the patient was completely recovered and discharged. Stool culture on follow-up visit was negative for* Shigella* confirming the complete recovery.

While the infant girl was being treated in our hospital, her 2.5-year-old brother was taken to the hospital on 12th July with major complaints of fever, bloody diarrhea, and abdominal cramps for the last three days. On examination, he was conscious but appeared pale and was dehydrated (grade II). His abdomen was tender and lower zone nondistended. No apparent hepatosplenomegaly was observed. His body temperature was 101°F, pulse rate was 120/minute, and respiratory rate was 32/minute. He was passing blood mixed thin stool frequently (9 times in the last twelve hours). Considering the previous case of acute dysentery in his younger sister, he was provisionally diagnosed as a case of acute diarrheal illness (probably dysentery). Necessary clinical and laboratory investigations along with appropriate clinical management were carried out with the antimicrobial regimen of Piperacillin and tazobactam (2 gm/24 hour) and 500 mg/24 hour metronidazole as empiric therapy was started as previous ESBL* Shigella* was documented in his sister. Zinc tablets (10 mg/24 hour), probiotic -bifilac (10 ml/24 hours), and oral rehydration solution (100 ml per stool episode) were also included in the therapy.

On ultrasonography, remarkable feature of intestinal intussusception between small bowels was noted. Considering it as a surgical emergency, immediate exploratory laparatomy was done to reduce the intussusceptions. Inflamed terminal ileum and caecum with ileocolic intussusception was noted during the surgery and it was reduced. In addition, inflamed appendix with mesenteric lymph nodes was noted and appendectomy was done.

On laboratory investigations, moderate decrease in hemoglobin concentration (10.7 gm%), with normal leucocytes and erythrocytes, was observed. Blood chemistry tests for urea, creatinine, and electrolytes were found on the normal range. Latex agglutination test for C-reactive protein was found strongly positive (++) indicating the systemic inflammation. Stool microscopy findings and cultural isolates along with its susceptibility were exactly similar to that of the previous case (Table 1). The patient was treated with intravenous Piperacillin tazobactam and metronidazole for 7 days and discharged on complete recovery. Similar to the previous case, follow-up culture of stool sample was negative for* Shigella* spp.

## 4. Genetic Characterization of ESBL by Polymerase Chain Reaction

**Figure 1:**
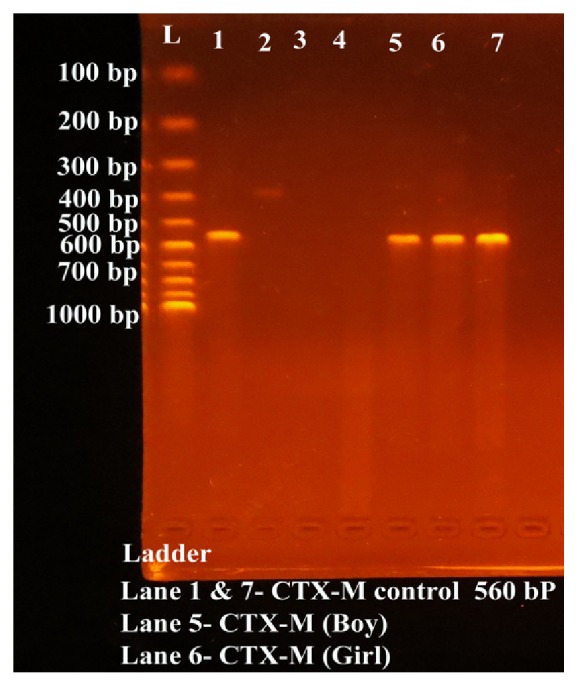
Electrophoretic bands of CTX-M ESBL after PCR.

Crude plasmid DNA was isolated from bacterial cells by using plasmid isolation kit (GeNei™) by using manufacturer instructions. Primers were obtained from GeNei, India, and they were used for identification of TEM, SHV, and CTX-M type ESBLs. The primer sequence is as shown in Table 2. Polymerase Chain Reaction (PCR) was carried out to detect the plasmid genes for SHV, CTX-M, and TEM type ESBL as previously described [13]. After PCR amplification, 2.5 *μ*l of each reaction was separated by electrophoresis in 1.5% agarose gel for 30 min at 100 V in 0.5 × TBE buffer. DNA was stained with ethidium bromide (1 *μ*g/ml) and the bands were detected using UV-transilluminator [Cleaver Scientific Ltd]. We found CTX-M type ESBL in both of the isolates tested confirming the ESBL Shigella in these two siblings (Figure 1).

## 5. Discussion

Worldwide, acute gastrointestinal infections including diarrhea are among the leading causes of morbidity and mortality among children, particularly in underdeveloped countries [2]. Poor access of safe water, inadequate sanitary conditions, lower literacy rate, and unavailability of health care facilities in the remote area are the major factors predisposing diarrheal illness among these countries [4, 13]. In our case, too, the unavailability of safe drinking water and lower socioeconomic status of the family might be associated with these* Shigella* infections in both siblings.

In both cases of this report, general symptoms of bacillary dysentery including abdominal discomfort, pallor, dehydration, and passage of blood tinged stool containing mucus have helped us to promptly investigate the clinical illness. Microscopic findings of erythrocytes and numerous nonlactose fermenting colonies grown on MacConkey agar further simulated the* Shigella* associated dysentery. In addition to this, abdominal ultrasonography was extremely useful in the second case for timely detection of intussusception in the small intestine. Similar cases of intestinal intussusception associated with* Shigella* spp. were reported from India [14] and USA [15, 16] among others.

Shigellosis or severe bacillary dysentery is disease of public health importance because it is associated with increased mortality and morbidity especially among the children of developing countries [17]. It is highly necessary to start a prompt and rational antibiotic regimen to minimize the clinical effects of severe dysentery and its complications [8]. Antimicrobial agents including fluoroquinolones and cephalosporins are the mainstay of therapy in severe cases of shigellosis but emerging reports of multidrug resistant strains from various parts of world made them less effective options [5]. Furthermore, the growing prevalence of ESBL producing strains among* Shigella* species is of immense concern [14].

This report represents the first case of ESBL producing* Shigella* strain associated with the clinical cases of shigellosis from the Himalayan country, Nepal. Previously, ESBL producing clinical strains of* Shigella* spp. has been reported from developing countries like India [5, 8, 14], Pakistan [18], Bangladesh [19, 20], Turkey [21], and others. In the past two decades both the isolation frequencies and the types of ESBLs have gradually increased. CTX-M, SHV, and TEM type ESBLs are being increasingly reported in the* Shigella* species around the globe [8]. The ESBLs are detected most commonly in* Klebsiella pneumoniae* and* Escherichia coli* but have been noted in other members of the Enterobacteriaceae family as well [22]. Macrolides such as azithromycin have been described as an alternative regime for empirical therapy for cases of severe dysentery particularly in children [5], but, in our cases, simultaneous resistance of fluoroquinolones and macrolides in the isolated strains of* Shigella* further limited therapeutic options. Frequent isolation of these multidrug resistant (MDR) and ESBL producing strains demands the higher antimicrobial regimens to be instituted. However, therapy with the intravenous broad spectrum drugs has greater economic as well as therapeutic constraints particularly in developing countries like Nepal where no any antimicrobial guidelines for specific infection are available.

CTX-M types ESBLs are plasmid-mediated *β*-lactamases having higher hydrolytic effect against cefotaxime. CTX-M-15 has been reported as common genotype of ESBL among* Shigella* isolates [23]. CTX-M type ESBL detected in both our cases could be the same genotype but we cannot analyze the further sequence. The molecular detection of various genes is the gold standard technique for identifying ESBL genes but routine screening with molecular tools is not practical in our country. Regular screening of all isolates strains for beta lactamase production and rational antibiotic prescription is highly necessary to curtail the spread of resistance determinants to other organisms of vicinity.

## 6. Conclusions

Detection of ESBL in* Shigella* has created the undeniable problem constricting the therapeutic choices for acute dysentery. Alongside, the mobile resistance determinants may transfer to wild strains of* Shigella* causing further dissemination of drug resistance. Timely diagnosis and appropriate antimicrobial regimen selection are vital in the management of invasive infections caused by* Shigella* strains.

## Figures and Tables

**Table 1:** Antibiogram of *Shigella flexneri* isolated from two siblings.

Antibiotics	Results
Ampicillin	*Resistant*
Azithromycin	*Resistant*
Cotrimoxazole	*Resistant*
Ceftazidime	*Resistant*
Cefixime	*Resistant*
Ciprofloxacin	*Resistant*
Gentamycin	*Resistant*
Piperacillin tazobactam	Sensitive
Ampicillin Sulbactam	*Resistant*
Imipenem	Sensitive
Meropenem	Sensitive

**Table 2:** Primers for the bla-CTX-M, bla-TEM, and bla-SHV genes.

Gene	Primers (5′-3′)	Amplicon size (bp)
SHV	F: 5′-GTCAGCGAAAAACACCTTGCC-3′	383 bp
	R: 5′-GTCTTATCGGCGATAAACCAG-3′	
TEM	F: 5′-GAGACAATAACCCTGGTAAAT-3′	459 bp
	R: 5′-AGAAGTAAGTTGGCAGCAGTG-3′	
CTX-M	F: 5′-GAAGGTCATCAAGAAGGTGCG-3′	560 bp
	R: 5′-GCATTGCCACGCTTTTCATAG-3′	
